# Use of Drug-Susceptibility Testing for Management of Drug-Resistant Tuberculosis, Thailand, 2004–2008

**DOI:** 10.3201/eid2003.130951

**Published:** 2014-03

**Authors:** Eugene Lam, Sriprapa Nateniyom, Sara Whitehead, Amornrat Anuwatnonthakate, Patama Monkongdee, Apiratee Kanphukiew, Jiraphan Inyaphong, Wanlaya Sitti, Navarat Chiengsorn, Saiyud Moolphate, Suporn Kavinum, Narin Suriyon, Pranom Limsomboon, Junya Danyutapolchai, Chalinthorn Sinthuwattanawibool, Laura Jean Podewils

**Affiliations:** US Centers for Disease Control and Prevention (US CDC), Atlanta, Georgia, USA (E. Lam, S. Whitehead, L.J. Podewils);; Thailand Ministry of Public Health (MOPH), Nonthaburi, Thailand (S. Nateniyom);; Thailand MOPH–US CDC, Nonthaburi (S. Whitehead, A. Anuwatnonthakate, P. Monkongdee, A. Kanphukiew, J. Danyutapolchai, C. Sinthuwattanawibool);; Office of Disease Prevention and Control 7, Ubon-ratchathani, Thailand (J. Inyaphong, W. Sitti);; Bangkok Metropolitan Health Administration, Bangkok Thailand (N. Chiengsorn);; Research Institute of Tuberculosis (RIT-Japan), Tokyo, Japan (S. Moolphate);; Tak Provincial Public Health Office, Tak, Thailand (S. Kavinum);; Chiang Rai Provincial Public Health Office, Chiang Rai, Thailand (N. Suriyon);; Phuket Provincial Public Health Office, Phuket, Thailand (P. Limsomboon)

**Keywords:** anti-tuberculosis treatment, laboratory testing, clinical management, treatment management, bacteria, TB, tuberculosis, drug resistance, rifampin-resistant tuberculosis, multidrug-resistant tuberculosis, MDR TB, Thailand, drug susceptibility testing, tuberculosis and other mycobacteria

## Abstract

In 2004, routine use of culture and drug-susceptibility testing (DST) was implemented for persons in 5 Thailand provinces with a diagnosis of tuberculosis (TB). To determine if DST results were being used to guide treatment, we conducted a retrospective chart review for patients with rifampin-resistant or multidrug-resistant (MDR) TB during 2004–2008. A total of 208 patients were identified. Median time from clinical sample collection to physician review of DST results was 114 days. Only 5.8% of patients with MDR TB were empirically prescribed an appropriate regimen; an additional 31.3% received an appropriate regimen after DST results were reviewed. Most patients with rifampin -resistant or MDR TB had successful treatment outcomes. Patients with HIV co-infection and patients who were unmarried or had received category II treatment before DST results were reviewed had less successful outcomes. Overall, review of available DST results was delayed, and results were rarely used to improve treatment.

Tuberculosis (TB), caused by the bacterium *Mycobacterium tuberculosis*, is a global public health issue; 8.6 million incident cases and 1.3 million deaths were attributed to TB in 2012 (*1*). A severe threat to TB control is the emergence of multidrug-resistant (MDR) TB: worldwide, there are an estimated 650,000 MDR TB cases (*1*). To manage MDR TB, the World Health Organization (WHO) recommends empirically basing treatment on the general drug-susceptibility testing (DST) pattern of the population for patients in settings with limited laboratory capacity or for patients with pending DST results. Once laboratory results become available, WHO recommends treatment modification, as needed, according to the DST results (*2*).

Algorithms have been proposed to assist with clinical decision-making, but a proper laboratory diagnosis remains the benchmark for informing an effective and suitable treatment regimen (*3*). New technologies to more quickly detect TB, including drug-resistant TB, have become increasingly available (*4*–*8*). These tests are in use throughout the world, potentially improving the clinical management of TB by informing clinicians of which drugs may be most effective for individual patients. However, because of delays in receiving laboratory results (*3*), clinicians may have adopted a convention of patient clinical management that relies on epidemiologic data, medical history, and clinical signs and symptoms. Continuation of ineffective treatment causes excess patient illness and increases the potential for drug resistance, relapse, death, and transmission of drug-resistant *M. tuberculosis* strains. Earlier initiation of proper therapy may therefore result in substantial cost savings (*9*). Thus, with these new diagnostic technologies has come the critical need to examine how test results are being used in the clinical management of TB patients.

In 2004, the Thailand Ministry of Public Health–US Centers for Disease Control and Prevention Collaboration implemented routine liquid culture and DST of clinical samples for all persons with a diagnosis of TB disease in 5 provinces participating in the Active TB Surveillance Network (*10*). Clinicians were provided orientation to the new diagnostic tests and their interpretation and limitations. Although these procedures had been implemented, the extent to which clinicians were using DST results to inform treatment decision making was not known. During 2004–2008, we conducted a retrospective chart review to 1) determine sociodemographic, clinical, and laboratory characteristics of persons with a diagnosis of rifampin (RIF)-resistant TB or MDR TB (i.e., resistance to RIF and isoniazid [INH]); 2) determine the timing and use of DST results; 3) determine the effect of DST results on treatment regimens used for RIF-resistant and MDR TB; and 4) determine the association between treatment regimen characteristics and treatment outcomes.

## Materials and Methods

### Study Population

For the evaluation, we selected patients with DST results demonstrating infection with RIF-resistant or MDR TB who were registered for TB treatment during October 2004–September 2008 at health facilities within the Thailand TB Active Surveillance Network (*10*). The surveillance network included 7 health centers in the Bangkok Metropolitan Area and government hospitals in Chiang Rai, Phuket, Ubon Rachathani, and Tak Provinces. Patients from health facilities operated by private practitioners, nongovernmental organizations, or facilities serving solely as referral centers that do not manage ongoing treatment and outpatient care were excluded. Patients with incomplete laboratory data (e.g., missing date of specimen collection or missing DST results) and those with non-TB mycobacterium infection or a change in diagnosis were also excluded.

### Data Collection and Laboratory Testing

Trained clinic staff retrospectively collected patient information from routine medical and laboratory records onto standardized forms. For each patient, we recorded dates for the following: specimen collection for DST, receipt of DST results at the clinic, and first clinic visit for patients after DST results became available. We also recorded the date of each clinic visit, all drugs and dosages included in treatment regimens, and all treatment changes throughout the course of treatment.

Sputum specimens from patients were cultured at a provincial government laboratory by using Lowenstein-Jensen solid culture and Mycobacterial Growth Indicator Tube (MGIT) liquid culture (BACTEC 960, Becton-Dickinson, Franklin Lakes, NJ, USA) according to standard procedures (*11*). Isolates were sent to the Bangkok or Thailand Ministry of Public Health National Reference Laboratory for identification and DST for first-line anti-TB drugs (i.e., streptomycin [STR], INH, RIF, and ethambutol [EMB]); Lowenstein-Jensen–based and MGIT-based methods were used for DST.

### Definitions and Treatment Regimens

Standard WHO definitions were used to categorize patients according to TB treatment history, site of TB infection, and treatment outcomes (*2*,*12*). Patients who completed treatment and those who were cured of TB were considered to have successful outcomes; patients for whom treatment failed and those who defaulted or died were considered to have poor treatment outcomes.

At the time this cohort of patients received a diagnosis and was clinically managed, national guidelines in Thailand recommended the use of standardized TB treatment regimens for MDR TB (*13*). These guidelines were not consistent with WHO guidelines (*2*); instead, they included only 3 months of a standard intensive-phase, injectable-based regimen and provided the option of using STR, rather than an aminoglycoside, as the injectable drug if the organism did not have documented STR resistance (*2*,*13*). Second-line drugs for MDR TB treatment were available to clinicians on request from a single source supported by the Department of Disease Control, Thailand Ministry of Public Health; the request process was not well standardized. For the purposes of this analysis, anti-TB treatment for MDR TB was considered appropriate if it was consistent with the Thailand national guidelines or if it was based on at least 3 drugs presumed to be effective according to the patient’s first-line DST results (*13*). At the time, there were no specific recommendations for treatment of RIF-resistant TB in Thailand, and treating physicians were not required to follow a specific standard for drug-resistant TB treatment.

### Data Analysis

We used frequencies and summary statistics to describe patient characteristics, DST patterns, DST turnaround times, and treatment regimens prescribed for patients. Characteristics were assessed by each category of drug-resistance (RIF-resistant or MDR TB) and for the total sample population. Baseline characteristics of patients included in the analysis and of those excluded from analysis were compared by using the Wilcoxon-Mann-Whitney test for continuous variables and the χ^2^ test for categorical variables; these tests were also used to compare RIF-resistant and MDR TB patient groups in the analytic sample.

We used log-binomial analysis to calculate the odds ratios (ORs) and 95% CIs to evaluate the association between baseline demographic and clinical factors and prescription of an inappropriate treatment among patients with MDR TB. The analytic sample for evaluating the association between treatment characteristics and treatment outcomes was restricted to patients with final treatment outcomes available (excluding patients who transferred out or who were still receiving treatment) at the time of analysis. We also used log-binomial regression analysis to calculate the OR and 95% CI for the association between characteristics of the treatment regimen and final treatment outcomes for patients with RIF-resistant or MDR TB. All models were initially age-adjusted, and other factors were chosen for inclusion in multivariate analyses if the p value was <0.20 in bivariate analysis or if there was epidemiologic plausibility or previously published evidence suggesting an association with treatment outcomes. Collinearity and effect modification were assessed for all variables in the multivariate models. Significance was considered at p<0.05. We used STATA version 10 (StataCorp, College Station, TX, USA) for all analyses.

## Results

### Patient and Clinical Characteristics

**Figure 1:**
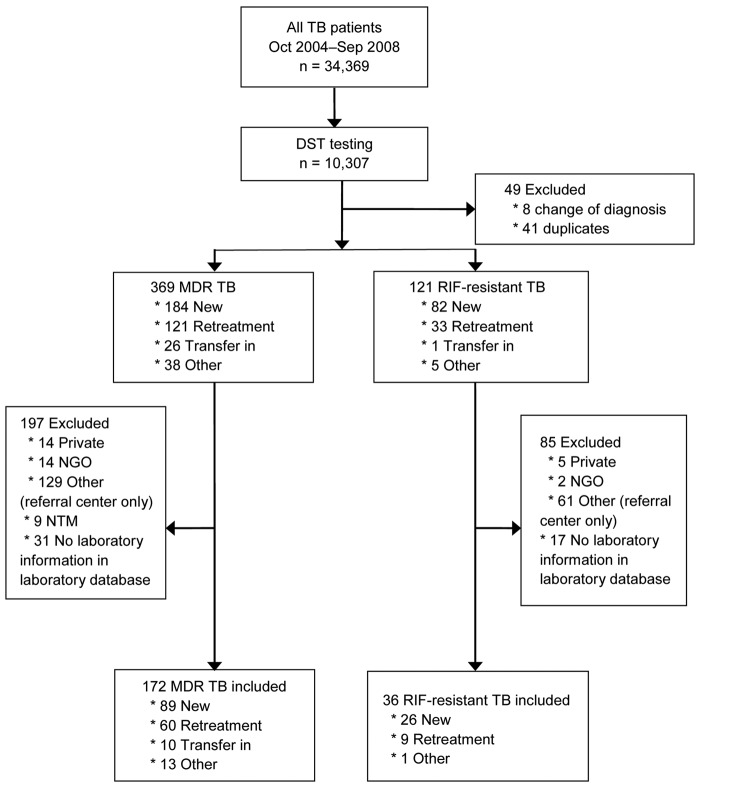
Selection of patients for an analysis of drug-susceptibility testing and management of drug-resistant tuberculosis, Thailand, October 2004–September 2008. Patients had rifampin- or multidrug-resistant tuberculosis and were from 5 Thailand provinces participating in the Thailand Active TB Surveillance Network. TB, tuberculosis; DST, drug-susceptibility testing; MDR, multidrug resistant; RIF, rifampin; Private, patient from private hospital; NGO, patient from nongovernmental hospital; NTM, nontuberculosis mycobacterium.

We identified a total of 490 patients as having TB that was RIF-resistant (n = 121) or MDR (n = 369) (Figure 1). Of these 490 patients, 208 (36 with RIF-resistant TB and 172 with MDR TB) were included in the evaluation. Patients with RIF-resistant TB who were excluded from analysis were substantially younger than those who were included in the analysis, and a substantially larger proportion of patients with HIV infection plus extrapulmonary or pulmonary and extrapulmonary disease were excluded from the RIF-resistant and MDR TB patient groups (Tables 1 and 2).

**Table 1:** Baseline demographic characteristics for patients with drug-resistant TB, Thailand, 2004–2008*

Characteristic	Patients with RIF-resistant TB, n = 121				Patients with MDR TB, n = 369				Patients with RIF-resistant vs. MDR TB, p value‡
	Included, n = 36	Excluded, n = 85	P value†		Included, n = 172		Excluded, n = 197	P value†	
Age, median (IQR), y	42 (34–58)	36 (29–48)	0.02		39 (29–50)		36 (28–47)	0.11	0.10
Sex									
M	22 (61.1)	55 (64.7)	0.71		117 (68.0)		128 (65.0)	0.58	0.42
F	14 (38.9)	30 (35.3)			55 (32.0)		68 (34.5)		
Data missing	0 (0.0)	0 (0.0)			0 (0.0)		1 (0.5)		
Marital status									
Married	22 (61.1)	39 (46.4)	0.32		93 (54.1)		85 (43.1)	0.001	0.06
Single/divorced/widowed	13 (36.1)	43 (51.2)			79 (45.9)		98 (50.0)		
Data missing	1 (2.8)	2 (2.4)			0 (0.0)		13 (6.6)		
Nationality									
Thai	33 (91.7)	76 (89.4)	0.70		144 (83.7)		170 (86.3)	0.49	0.22
Not Thai	3 (8.3)	9 (10.6)			28 (16.3)	27 (13.7)			

*Patients were from the Thailand TB Active Surveillance Network and were either included or not included in the current study. Data are no. (%) unless otherwise indicated. TB, tuberculosis; RIF, rifampin; MDR, multidrug-resistance; IQR, interquartile range. †p values reflect comparison between included and excluded patients in each TB drug resistance group. ‡p values reflect comparison between included RIF-resistant patients and MDR TB patients.

**Table 2:** Baseline clinical characteristics for patients with drug-resistant TB, Thailand, 2004–2008*

Characteristic	No. (%) patients with							Patients with RIF-resistant vs. MDR TB, p value‡
	RIF-resistant TB, n = 121				MDR TB, n = 369			
	Included, n = 36	Excluded, n = 85	P value†		Included, n = 172	Excluded, n = 197	P value†	
Case status								
New	26 (72.2)	56 (65.9)	0.64		89 (51.7)	95 (48.2)	0.51	0.17
Retreatment after relapse	6 (16.7)	19 (22.4)			25 (14.5)	24 (12.2)		
Retreatment after failure	2 (5.6)	1 (1.2)			21 (12.2)	19 (9.6)		
Retreatment after default	1 (2.8)	4 (4.7)			14 (8.1)	18 (9.1)		
Transfer in	0 (0.0)	1 (1.2)			10 (5.8)	16 (8.1)		
Other	1 (2.8)	4 (4.7)			13 (7.6)	25 (12.7)		
Site of TB								
Pulmonary	33 (91.7)	61 (71.8)	0.05		160 (93.0)	162 (82.2)	0.008	0.69
Extrapulmonary	2 (5.6)	13 (15.3)			5 (2.9)	15 (7.6)		
Both	1 (2.8)	11 (12.9)			7 (4.1)	20 (10.2)		
Cough >2 wk								
No	11 (30.6)	39 (45.9)	0.22		40 (23.3)	61 (31.0)	<0.001	0.36
Yes	25 (69.4)	45 (52.9)			132 (76.7)	123 (62.4)		
Data missing	0 (0.0)	1 (1.2)			0 (0.0)	13 (6.6)		
Smear status								
Negative	4 (11.1)	11 (12.9)	0.44		26 (15.1)	26 (13.2)	0.57	0.71
Positive	30 (83.3)	63 (74.1)			140 (81.4)	160 (81.2)		
Data missing	2 (5.6)	11 (12.9)			6 (3.5)	11 (5.6)		
Chest radiograph								
Normal	0 (0.0)	4 (4.7)	0.27		4 (2.3)	10 (5.1)	0.17	0.79
Abnormal, no cavity	22 (61.1)	41 (48.2)			109 (63.4)	104 (52.8)		
Abnormal, with cavity	8 (22.2)	29 (34.1)			34 (19.8)	48 (24.4)		
Data missing	6 (16.7)	11 (12.9)			25 (14.5)	35 (17.8)		
HIV status								
Negative	23 (63.9)	29 (34.1)	0.004		112 (65.1)	92 (46.7)	0.001	0.17
Positive	13 (36.1)	46 (54.1)			47 (27.3)	75 (38.1)		
Data missing	0 (0.0)	10 (11.8)			13 (7.6)	30 (15.2)		
Outcome								
Treatment success								
TB cured	20 (55.6)	26 (30.6)	0.25		51 (29.7)	33 (16.8)	0.06	0.07
Treatment completed	5 (13.9)	21 (24.7)			25 (14.5)	36 (18.3)		
Poor outcome								
Treatment failed	1 (2.8)	5 (5.9)			31 (18.0)	29 (14.7)		
Patient defaulted	3 (8.3)	10 (11.8)			17 (9.9)	28 (14.2)		
Patient died	5 (13.9)	12 (14.1)			30 (17.4)	40 (20.3)		
Transferred out	2 (5.6)	9 (10.6)			15 (8.7)	23 (11.7)		
Ongoing treatment	0	2 (2.4)			3 (1.7)	8 (4.1)		

*Patients were from the Thailand TB Active Surveillance Network and were either included or not included in the current study. TB, tuberculosis; RIF, rifampin; MDR, multidrug-resistance. †p values reflect comparison between included and excluded patients in each TB drug resistance group. ‡p values reflect comparison between included RIF-resistant and MDR TB patients.

Among the 172 patients with MDR TB included in the analysis, 89 (51.7%) were new TB patients, 60 (34.9%) were retreatment patients, and 10 (5.8%) transferred in from another facility (Table 2). Among the 36 patients with RIF-resistant TB, 26 (72.2%) had newly diagnosed infection and 9 (25.0%) were retreatment patients (Table 2). The median age of patients with RIF-resistant and MDR TB was 42 years (interquartile range [IQR] 34–58) and 39 years (IQR 29–50), respectively (Table 1). Overall, baseline characteristics were comparable for patients with RIF-resistant and MDR TB. Most patients in both groups were male, married, and HIVseronegative.

### Drug-Susceptibility Patterns

Of the 36 patients with RIF-resistant TB, 28 (77.8%) had resistance to RIF alone. Another 4 (11.1%) also had resistance to RIF and STR; 2 (5.6%) had resistance to RIF and EMB; and 2 (5.6%) had resistance to RIF, EMB, and STR.

Among the 172 patients with MDR TB, 69 (40.1%) had resistance to only INH and RIF. Another 13 (7.6%) had resistance to INH, RIF, and EMB; 55 (32.0%) had resistance to INH, RIF, and STR; and 35 (20.3%) had resistance to STR and EMB.

### Turnaround for Drug-Susceptibility Testing Results

The median time from collection of patient sputum samples to clinic receipt of DST results was 97.5 days (IQR 66–133.3) (Table 3). The median time from clinic receipt of DST results to physician review of results at the first post-DST visit was 7 days (IQR 1–21). Overall, the median time from sputum collection to the first physician review of the DST result was 109.5 days (IQR 73–150). Patients with MDR TB had longer diagnostic turnaround times than patients with RIF-resistant TB.

**Table 3:** Diagnostic turnaround for DST results for 130 RIF-resistant and MDR TB patients, Thailand 2004–2008*

Turnaround variable	Median (IQR), d		
	RIF-resistant TB (n = 18)	MDR TB (n = 112)	Total RIF-resistant and MDR TB
Time from sputum collection to clinic receipt of results	75.0 (49.0–112.0)	100.0 (67.3–137.5)	97.5 (66.0–133.3)
Time from clinic receipt of results to review by physician†	9.0 (0.8–16.5)	7.0 (1.0–21.8)	7.0 (1.0–21.0)
Time from sputum collection to result review by physician†	83.0 (53.0–130.3)	111.0 (77.3–153.3)	109.5 (73.0–150.0)

*The 130 TB patients represented here were among 208 patients from 5 Thailand provinces participating in the Thailand Active TB Surveillance Network. Calculations were restricted to patients who had complete information for sputum collection date, date of receipt of DST at the clinic, and date of the first physician visit after availability of DST results. Three MDR TB patients were missing initial sputum collection date, 14 RIF-resistant and 41 MDR TB patients were missing date of receipt of DST results at the clinic, and 16 RIF-resistant and 47 MDR TB patients were missing date of first clinic visit following receipt of DST results. DST, drug susceptibility test; RIF, rifampin; MDR, multidrug-resistant; TB, tuberculosis; IQR, interquartile range. †Represents first post-DST clinic visit.

### TB Treatment

Of the 172 patients with MDR TB included in the analysis, 10 (5.8%) were initially prescribed an appropriate treatment regimen, and 51 (29.7%) were prescribed appropriate treatment at some point during the treatment course. Forty-one patients with MDR TB and 13 with RIF-resistant TB were not eligible for treatment changes after clinic results became available: 31 of these MDR TB and all 13 patients with RIF-resistant TB had treatment outcomes before the return of DST results, and the other 10 patients with MDR TB were empirically treated with appropriate drugs.

**Figure 2:**
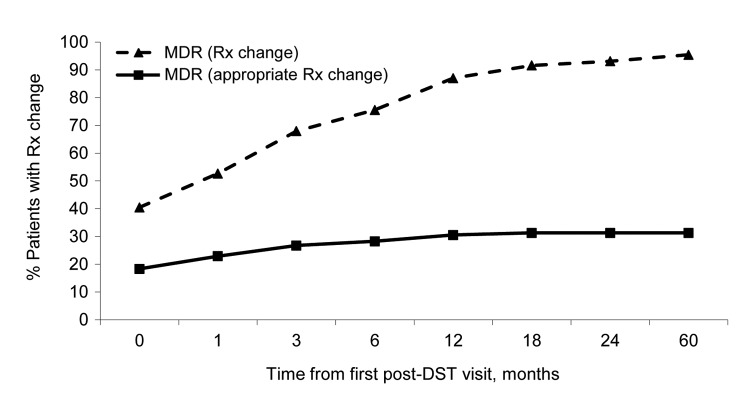
Percentage of MDR-TB patients who were eligible for a treatment regimen change (n = 131) who received a change, according to time from the first review of DST result by the physician, TB Active Surveillance Network, Thailand 2004–2008. Rx, prescription treatment. DST, drug-susceptibility testing; MDR, multidrug-resistant TB.

Of the remaining 131 patients with MDR TB, 37 (28.2%) never had a treatment change, 53 (40.5%) had a treatment change at the first clinic visit following availability of DST results, and 41 (31.3%) had changes made later in the treatment course. Of the 53 patients with a change made at the first post-DST visit, 24 (45.3%; 18.3% of those eligible for change) were prescribed on appropriate regimens; 9 of the 24 changes were in accord with the national treatment guidelines, and the 15 other changes were to >3 drugs presumed to be effective (Figure 2). Of the remaining 29 MDR TB patients who had a regimen change at the first post-DST visit, 3 were changed to a category I regimen and 26 were placed on a nonstandard second-line treatment combination. By 3 months after the first physician review of the DST results, 51.7% of these 131 patients with MDR TB received changes to their treatment plan, of which ≈20% were appropriate changes. At 12 months and onward after the first physician review of DST results, ≈85% of patients with MDR TB had changes to their treatment, of which ≈30% were classified as appropriate adjustments.

Of the 23 patients with RIF-resistant TB who were eligible for treatment changes, 4 (17.4%) had a treatment change at the first post-DST visit, and the remaining 19 (82.6%) patients had changes made during subsequent post-DST clinic visits. Of the 4 patients with RIF-resistant TB who received regimen changes at the first post-DST visit, 2 were changed to a nonstandard first-line treatment combination (INH + pyrazinamide + EMB + STR or INH + EMB), 1 was changed to a nonstandard second-line treatment combination (INH + EMB + ofloxacin), and 1 discontinued treatment due to hepatic cirrhosis. Figure 2 is restricted to MDR TB patients because there was no written guideline on appropriate treatment of patients with RIF-resistant TB in Thailand during the study period; therefore, we were unable to differentiate between appropriate and inappropriate changes for the patients with RIF-resistant TB.

### Baseline Factors and Appropriateness of Treatment

Examination of baseline factors associated with prescription of an inappropriate treatment for MDR TB case-patients indicated that retreatment patients were significantly more likely than new patients to be prescribed an inappropriate regimen (age-adjusted OR 2.6, 95% CI 1.0–6.3; p = 0.04) (Table 4). A significant association was not identified between the time delay between sputum collection and the first clinic visit following availability of DST results and whether patients were prescribed appropriate regimens.

**Table 4:** Association between baseline sociodemographic and clinical characteristics and prescription of an inappropriate MDR TB treatment regimen for 172 MDR TB patients 2004–2008*

Characteristic	Univariate OR (95% CI)	p value	Multivariate OR (95% CI)†	p value
Age, y				
<45	Reference			
>45	0.54 (0.2–1.2)	0.13		
Sex				
Male	Reference			
Female	0.74 (0.3–1.8)	0.49		
Marital Status				
Married	Reference			
Single/divorced/widowed	0.44 (0.2–1.0)	0.06		
Nationality				
Thai	Reference			
Non-Thai	1.0 (0.4–3.0)	0.95		
Case status				
New	Reference		Reference	
Retreatment	2.4 (1.0–5.9)	0.05	2.6 (1.0–6.3)	0.04
Transfer in/other	2.8 (0.9–8.7)	0.08	2.9 (0.9–9.3)	0.07
Site of TB				
Pulmonary	Reference			
Extrapulmonary or both	0.41 (0.1–3.3)	0.40		
Cough >2 wk				
No	Reference			
Yes	1.6 (0.6–4.6)	0.35		
Smear status				
Negative	Reference			
Positive	6.5 (0.8–50.2)	0.07		
Cavitation seen on chest x-ray				
No	Reference			
Yes	0.40 (0.1–1.4)	0.16		
HIV status				
Negative	Reference			
Positive	0.38 (0.1–1.2)	0.09		
Resistance pattern				
INH + RIF	Reference			
INH + RIF + EMB or INH + RIF + STR	0.80 (0.3–2.1)	0.65		
INH + RIF + EMB + STR	2.1 (0.8–5.6)	0.13		
Delay from sputum collection to firs post-DST visit, d				
35–74	Reference			
75–112	1.3 (04–4.6)	0.70		
113–155	1.8 (0.5–6.2)	0.38		
>155	1.0 (0.3–3.8)	0.97		

*The patients were from 5 Thailand provinces participating in the Thailand Active TB Surveillance Network. OR, odds ratio; MDR, multidrug resistant; TB, tuberculosis; INH, isoniazid; RIF, rifampin; EMB, ethambutol; STR, streptomycin; DST, drug-susceptibility testing. †Adjusted for age as a continuous variable.

### Patient Group and Treatment Outcome

Twelve patients (2 with RIF-resistant TB, 10 with MDR TB) with treatment outcomes that indicated they had transferred out and 2 patients with MDR TB who were still receiving treatment at the time of the evaluation were excluded from the final analytic sample (final n = 194; 34 RIF-resistant TB and 160 MDR TB cases). Treatment success was slightly greater among the RIF-resistant TB group than the MDR TB group (76.5% vs. 60.6%, p = 0.08). Patients who were not married (adjusted OR 2.3, 95% CI 1.2–4.6; p = 0.01), who were HIV positive (adjusted OR 2.2, 95% CI 1.1–4.4; p = 0.04), and who received category II treatment before receiving DST results (adjusted OR 2.6, 95% CI 1.1–6.4; p = 0.05) had poorer treatment outcomes (Appendix, Table 5). In the analysis restricted to patients with MDR TB, receiving inappropriate treatment was not significantly associated with poor treatment outcomes (OR = 0.77, 95% CI 0.3–1.8; p = 0.55).

**Table 5, Appendix:** Association between sociodemographic, clinical, and treatment characteristics and poor treatment outcome among 194 RIF-resistant and MDR TB patients, Thailand 2004–2008*

Characteristic	Poor outcome/total outcomes†	Analysis, odds ratio (95% CI)				
		Univariate	p		Multivariate‡	p
Age, y						
<45	28/76 (36.8)	Reference				
>45	43/118 (36.4)	0.98 (0.5–1.8)	0.96			
Sex						
M	47/126 (37.3)	Reference				
F	24/68 (35.2)	0.92 (0.5–1.7)	0.78			
Marital status						
Married	31/106 (29.3)	Reference			Reference	
Single/divorced/widowed	40/87 (46.0)	2.1 (1.1–3.7)	0.02		2.3 (1.2–4.6)	0.01
Nationality						
Thai	57/164 (34.8)	Reference				
Not Thai	14/30 (46.7)	1.6 (0.7–3.6)	0.22			
Case status						
New	38/111 (34.2)	Reference				
Retreatment	27/64 (42.2)	1.4 (0.7–2.6)	0.30			
Transfer in/other	6/19 (31.6)	0.89 (0.3–2.5)	0.82			
Site of TB						
Pulmonary	65/179 (36.3)	Reference				
Extrapulmonary or both	6/15 (40.0)	1.2 (0.4–3.4)	0.78			
Cough >2 wk						
No	21/49 (42.9)	Reference				
Yes	50/145 (34.5)	0.70 (0.4–1.4)	0.29			
Smear status						
Negative	9/27 (33.3)	Reference				
Positive	60/159 (37.7)	1.2 (0.5–2.9)	0.66			
Cavitation seen on chest x-ray						
No	48/127 (37.8)	Reference				
Yes	14/37 (37.8)	1.0 (0.5–2.1)	1.0			
HIV status						
Negative	37/125 (29.6)	Reference			Reference	
Positive	28/57 (49.1)	2.3 (1.2–4.4)	0.01		2.2 (1.2–4.6)	0.04
Resistance pattern						
RIF	8/34 (23.5)	Reference				
MDR	63/160 (39.4)	2.1 (0.9–5.0)	0.09			
DOT						
Health care worker	21/65 (32.3)	Reference				
Family/other	45/112 (40.2)	1.4 (0.7–2.7)	0.30			
None	5/17 (29.4)	0.87 (0.3–2.8)	0.82			
Category II treatment						
None	50/155 (32.3)	Reference			Reference	
Pre-DST only	15/26 (57.7)	2.9 (1.2–6.7)	0.02		2.5 (1.1–6.4)	0.05
Post-DST/ full treatment course	4/9 (44.4)	1.7 (0.4–6.5)	0.45		2.8 (0.7–11.6)	0.16
Delay from sputum collection to first post-DST visit, d						
35–74	10/33 (30.3)	Reference				
75–112	5/31 (16.1)	0.44 (0.1–1.5)	0.19			
113–155	8/36 (22.2)	0.66 (0.2–1.9)	0.44			
>155	7/32 (21.9)	0.64 (0.2–2.0)	0.44			

*Patients were from the Thailand TB Active Surveillance Network and were restricted to those from the current study for whom final treatment outcomes were available (excluding transferred out, still receiving treatment). RIF, rifampin; MDR TB, multidrug-resistant tuberculosis; DOT, directly observed treatment; DST, drug-susceptibility testing.  †Poor treatment outcome included treatment failure, default, and death. Data are no. patients with poor outcome/no. patients total unless otherwise specified. ‡Adjusted for age as a continuous variable.

## Discussion

This evaluation revealed that most treatment regimens assigned to patients with RIF-resistant or MDR TB in selected areas of Thailand were not based on DST results. Less than one third of patients with MDR TB received appropriate treatment, and patients who had previously received treatment for TB were >2 times more likely to be prescribed an inappropriate treatment regimen. When DST results were available, treatment changes did not necessarily reflect nationally recommended standard regimens for drug-resistant TB or the resistance profile of the infecting TB strain. In some cases, physicians probably did not change to second-line treatment because of the clinical condition of the patient; only 16% of patients overall had smear-positive test results at month 5 (data not shown). Persistence of smear-negative test results among identified MDR TB cases has been cited as a reason for not changing to a standardized MDR TB treatment regimen; other reasons have included patient loss to follow-up, patient death, and patient refusal to change treatment (*14*). A study evaluating the influence of the microscopic observation drug susceptibility (MODS) assay, which allows for determination of drug susceptibility directly from sputum in just 7–10 days, on the clinical management of TB patients also reported that even when indicated, appropriate treatment regimen changes were not always made (*15*). MDR TB treatment is highly decentralized in Thailand, and some clinicians may not have been familiar with treatment guidelines.

The median delay from the time of sputum collection to the time DST results were available at the clinic exceeded 14 weeks, and further delays were noted between availability of results and a clinical encounter. During the evaluation period, MGIT liquid culture was used for diagnosis of TB and of drug resistance; the turnaround time for culture results is generally 4–6 weeks (*4*). Other studies have also demonstrated the effect of clinic delays on TB management, even when laboratory results are available in a timely manner (*9*,*14*,*16*). The time interval for diagnosing RIF-resistant and MDR TB in this evaluation was longer than expected, probably due to constraints with specimen transport, laboratory capacity, and administrative delays in providing results to clinics. In addition, MDR TB result reporting was further delayed because the implications for regimen change were considered more serious for MDR TB than for RIF-resistant TB; the reference laboratories tended to hold MGIT-based DST results until they were confirmed by solid culture. Our findings highlight these other sources of delay beyond those intrinsic to a given assay as pivotal for ensuring the benefits of rapid diagnostic technologies.

In this evaluation, patients with MDR TB were more likely to receive an inappropriate initial treatment regimen if they were a retreatment patient rather than a new patient. This finding suggests that retreatment cases should be prioritized when considering the application of rapid diagnostic technologies, and actions should be taken to expedite the transport and testing of specimens and reporting results to the clinician. In addition, patients who initially received category II treatment were significantly more likely to default, fail treatment, or die. This finding is consistent with those of previous studies demonstrating the association between category II treatment and poor outcomes and the growing body of evidence advocating for the elimination of the category II retreatment regimen (*17*–*21*).

Multiple studies have reported high rates of treatment success among patients prescribed individualized regimens tailored to DST results (*22*–*27*). In a recent meta-analysis of 33 studies in 20 countries evaluating MDR TB treatment outcomes, individualized treatment had higher treatment success compared with standardized regimens based on local drug-susceptibility patterns (64 vs. 54%), although the difference was not statistically significant (*28*). In our study, the lack of direct association between the appropriateness of the treatment regimen and treatment outcomes among patients with MDR TB may have been due to the small number of patients prescribed appropriate regimens during the treatment course, or it may be that treatment decisions based on other clinical factors were more pertinent to determining outcomes.

Our analysis has limitations. First, patients were excluded if they had incomplete laboratory or clinic data, including patients for whom the date of specimen collection or receiving or reviewing the DST results at the clinic were not recorded. However, we do not have any indication that the omission of this information was systematic. Second, it is possible that our conclusions are not representative of all patients in Thailand with RIF-resistant or MDR TB. We noted that a higher proportion of patients in the excluded group than in the analytic sample were HIV positive and had extrapulmonary TB; this disproportion possibly occurred because of our inclusion requirement of complete laboratory data, and the microbiological yield from these 2 clinical groups is often low. In addition, one referral facility with a high proportion of HIV-associated TB cases was excluded because as a facility providing episodic tertiary consultation, they rarely have complete diagnostic and treatment data for patients. Last, the data for our study were abstracted from medical charts and routine surveillance not intended for specific research purposes; it is possible that some drug adjustments were not identified. Because of the retrospective study design, detailed information on factors considered in clinical decision-making and treatment prescriptions for patients was not available if it was not explicitly documented in the medical records. DST is only one component considered in prescribing treatment; the patient’s clinical status and risks involved with alternate drugs are also key factors. The long delays in obtaining DST findings may result in a heavier reliance on clinical factors for prescribing decisions.

Future research that identifies reasons for the low utilization of laboratory results when prescribing anti-TB therapy will help to develop interventions that can facilitate optimal treatment for drug-resistant TB. Furthermore, evaluation is needed to determine where and why delays unrelated to assay result turnaround times occurred; such delays may occur during specimen transport or processing, or they may be related to the timing of clinic notifications or the review of results by clinic physicians. Physicians’ knowledge of the national guidelines and treatment algorithms as well as their ability to interpret and use DST results to improve treatment regimens should be assessed. Assessment of DST result uptake in other health sectors (e.g., private practice, nongovernmental organizations, and referral centers) would be informative because several participants who were at high risk for treatment failure (i.e., patients with HIV infection or extrapulmonary disease) were excluded from the current study.

In conclusion, utilization of DST results in the clinical management of patients with RIF-resistant or MDR TB was poor in Thailand during 2004–2008. Since the time of this evaluation, access to second-line drugs has improved in Thailand: the request process has been streamlined and standardized, and the national treatment guidelines have been clarified and strengthened and disseminated to clinicians. Attention to the DST reporting system has also reduced delays somewhat. These factors will need to be considered in assessing the effect of more rapid molecular testing methods.
